# Individual-based population genomics reveal different drivers of adaptation in sympatric fish

**DOI:** 10.1038/s41598-020-69160-2

**Published:** 2020-07-29

**Authors:** Héctor Torrado, Carlos Carreras, Núria Raventos, Enrique Macpherson, Marta Pascual

**Affiliations:** 1 Centre D’Estudis Avançats de Blanes (CEAB-CSIC), Car. Acc. Cala St. Francesc 14, 17300 Blanes, Girona Spain; 2 Department de Genètica, Microbiologia i Estadística and IRBio, Universitat de Barcelona, Av. Diagonal 643, 08028 Barcelona, Spain

**Keywords:** Marine biology, Ecological genetics, Evolutionary ecology

## Abstract

Connectivity and local adaptation are two contrasting evolutionary forces highly influencing population structure. To evaluate the impact of early-life traits and environmental conditions on genetic structuring and adaptation, we studied two sympatric fish species in the Western Mediterranean Sea: *Symphodus tinca* and *S. ocellatus*. We followed an individual-based approach and measured early-life history traits from otolith readings, gathered information on environmental variables and obtained genome-wide markers from genotyping-by-sequencing (GBS). The two species presented contrasting population structure across the same geographic gradient, with high and significant population differentiation in *S. ocellatus*, mostly determined by oceanographic fronts, and low differentiation and no front effect in *S. tinca*. Despite their different levels of genetic differentiation, we identified in both species candidate regions for local adaptation by combining outlier analysis with environmental and phenotypic association analyses. Most candidate loci were associated to temperature and productivity in *S. ocellatus* and to temperature and turbulence in *S. tinca* suggesting that different drivers may determine genomic diversity and differentiation in each species. Globally, our study highlights that individual-based approach combining genomic, environmental and phenotypic information is key to identify signals of selection and the processes mediating them.

## Introduction

Population structure is highly influenced by the level of connectivity among localities, driven by dispersal potential and barriers to gene flow, but also shaped by local adaptation. In this context, environmental conditions can impose spatially differential genomic selective pressures on phenotypic traits and their combined study can allow to forecast the species adaptive capacity^1^. The use of environmental information coupled with the genetic structure of species can be essential for understanding how they are adapted to their habitats^2^. Some studies have investigated the relationship between local adaptation and environmental conditions in several marine species with temperature, salinity and productivity values being important factors affecting different biological processes and genetic differentiation at the population level^3–6^. For instance, thermal gradients observed at sea can explain the distributional patterns of numerous species, their diversification processes and the potential influence of climate changes^7^.

Population genomic studies are fundamental to identify patterns of genetic structure and the processes determining them. Genotyping-by-sequencing (GBS)^8^, provides a cost-effective approach for population genomic studies on non-model species. This methodology provides high density of markers that may allow identifying non-neutral genomic signatures related to adaptation processes, with the potential for annotation^6,9^. The assessment of genomic signals of adaptation on non-model organisms is usually performed in two different ways, outlier analyses (OAs) and genotype-environment association analyses (EAAs)^2^. On one hand, OAs are based on the search of genome-wide markers showing outlier values of genetic differentiation (F_ST_) among the studied populations^10^. On the other hand, EAAs using population genomic data combined with environmental data have been successfully used to discover polymorphisms involved in adaptation to environmental conditions^3, 6^. However, individual-based information could be used to further refine those associations. Individual-based information is used in genome-wide association studies (GWAS), which can identify signals of adaptation to both environmental conditions (EAAs) and phenotypic traits through phenotype association analysis (PAAs). This methodology has proven to be a powerful tool to study interesting phenotypic traits and adaptation in model species such as humans, plants and captive fish^11–13^. While the use of individual-based information is common in model species, the application in wild populations is rare and has been only used in a few studies combined with environmental data information^14^. Up to now, there is a lack of individual-based studies in natural marine populations, preventing an accurate knowledge of the relationships between genomic data, environment and early life phenotypes in determining population structure in environmental gradients. This approach can be undertaken in species were individual based information during dispersal phases can be obtained, such as benthic fishes.

In numerous Mediterranean benthic fishes, the pelagic larval stage allows dispersal and maintains connectivity between populations, whereas adults are territorial and present a high site fidelity^15–18^. The length of the pelagic larval duration (PLD) can not only affect dispersal but also other fitness characteristics such as larval growth, size at settlement and mortality rate, modulating the year-class strength in numerous species^19,20^. Moreover, environmental conditions can have major effects during the larval and settlement periods, where genetic and phenotypic polymorphism can be affected by selection and/or stochastic factors^15^. Fortunately, in most fishes, individual-based information on both phenotypic and environmental early-life variables can be obtained from otoliths (bones in the inner part of the ear) and has been successfully combined with genomic data to infer fine-scale dispersal^21^. Therefore, the study of individually based genomic variation associated to early-life history traits across geographic gradients in sympatric fish can provide insights into the adaptive potential of species while considering both connectivity and selection.

Here we use an individual-based approach to evaluate the phenotypic and environmental drivers shaping population genomic structure in two congeneric fish, *Symphodus tinca* and *S. ocellatus,* that are sympatric across a geographic/environmental gradient in the Western Mediterranean Sea. We selected two closely related species to ensure that any difference found between the two species on the drivers shaping population structure is not the result of a phylogenetic signal of divergent lineages, but a species-specific differential adaptation signal. On the other hand, adaptation signals on marine organisms may change depending on the environmental gradient present on the sampling sites and the scale of analysis^6^. For this reason, a sympatric distribution is key to check for a differential adaptation strategy, as both species are going to be under the same environmental gradient and face the same oceanographic fronts. We analysed 303 individuals from these two species with genotyping by sequencing, measured from otolith readings some individualized key early-life history traits (e.g. date of hatching, pelagic larval duration, size at settlement, growth rate) and gathered information of environmental variables during their pelagic larval duration (e.g. surface temperature, turbulence, and productivity). In order to test if phenotypic and environmental variables differentially influence the population structure in these two species, we (1) gathered individual-based information on early-life phenotypic and environmental variables, (2) assessed population differentiation, (3) identified loci putatively under selection using different methodological approaches and (4) evaluated the effect of phenotypic and environmental factors in structuring populations across geographic gradients. We expected to find similar associations and genomic structuring for both species due to their similar distribution and phylogenetic closeness. Nevertheless, considering their differences in reproduction times, temporal environmental variation and behaviour could change population connectivity and the drivers of population structure.

## Results

### Phenotypic and environmental analyses

**Figure 1:**
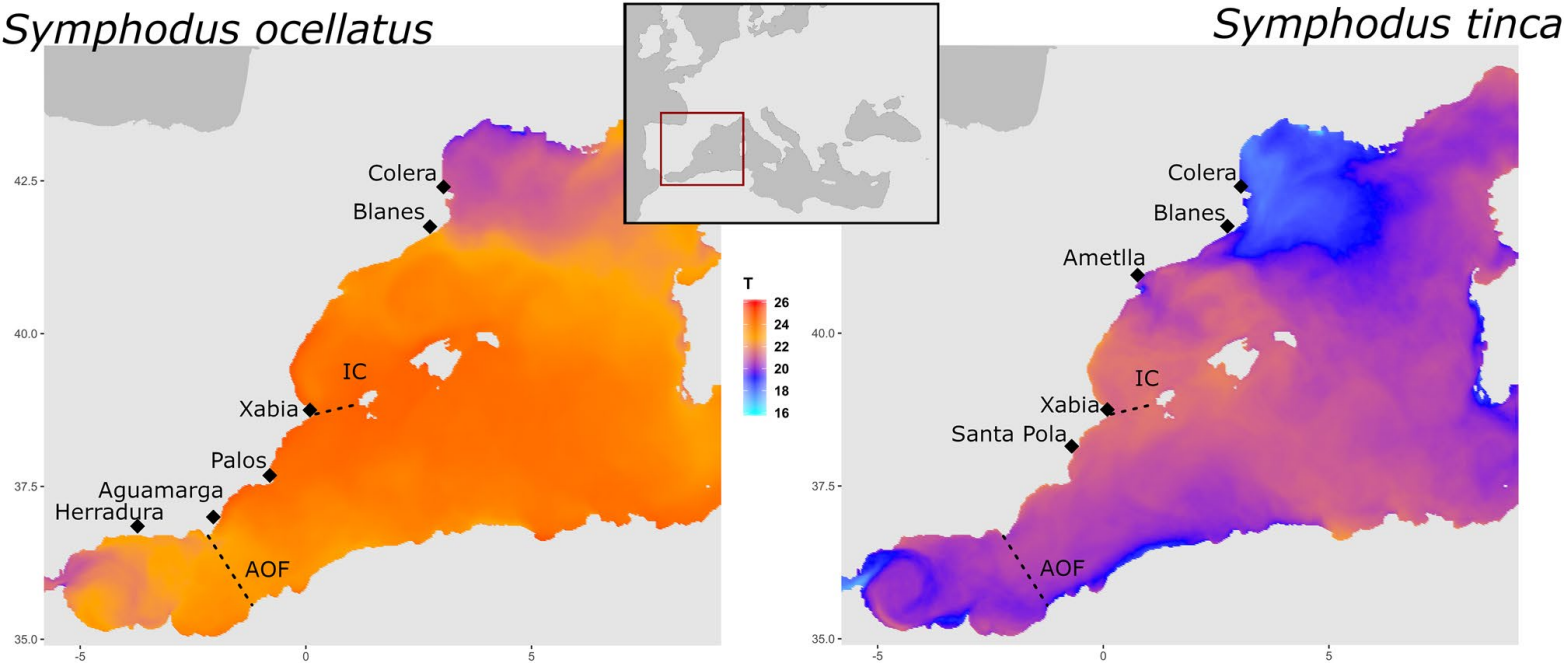
Map of the sampled locations in the Western Mediterranean for *Symphodus ocellatus* and *S. tinca*. Dashed lines indicate oceanographic discontinuities: Ibiza channel (IC) and Almeria-Oran front (AOF). The colour gradient of the sea identifies the mean sea surface temperature (ºC) during the larval period of each species. The reduced grey map shows the location of our study area within the Mediterranean Sea. Data obtained from Western Mediterranean OPerational forecasting system (WMOP)^63^.Maps were plotted using the R^60^ packages ‘ncdf4’^61^ and ‘ggplot2’^62^.

We obtained reliable information on early-life history traits from otolith reading (Figure S1) for 93% and 89% of the juvenile individuals from most of the localities in *S. ocellatus* and *S. tinca*, respectively (Fig. 1, Table 1). With these data we acquired environmental and phenotypic individually based values (Table 2). Significant differences among sampling locations were detected with PERMANOVA for all phenotypic and environmental variables with three exceptions: PLD growth rate and Hatching date in *Symphodus ocellatus* and PLD in *S. tinca* (Table S1). The temperature during the larval stage was lower in northern localities for both species, even considering a delay on the hatching date in some of these localities to encounter warmer dates (Table 2). For *S. ocellatus,* individuals from the colder areas (Fig. 1), the Alboran Sea and the two northernmost localities, delayed hatching date and thus experienced milder temperatures during PLD than otherwise expected (Table 2). In the case of *S. tinca*, the individuals of colder localities (Fig. 1) did not always delay their hatching date. For instance, in Blanes, individuals hatched early in the season and thus experienced low temperatures and had longer PLD (Table 2). The information of multiple variables was synthesized with principal component analysis (PCA). The PCA yielded several components for the phenotypic (PC) and environmental (EC) variables, with different contribution from each variable, and overall explaining a large proportion of the variance (Table S2).

**Table 1:** Sampling information and population diversity values for the locations of *Symphodus ocellatus* and *S. tinca*. Sampling date, Number of genotyped individuals (N) and in parentheses the number of juvenile individuals with otoliths information, Allelic richness (Ar), Mean observed (H_o_) and expected (H_e_) heterozygosities, Inbreeding coefficient (F_IS_). All F_IS_ values are significant. The names of localities in parentheses indicate the toponymal names of the corresponding localities. Diversity values were obtained from the haplotype loci datafiles.

Species	Location	Sampling date	N	Ar	Ho	He	F_IS_
*Symphodus ocellatus*	Colera 42°24′26.9"N 3°09′23.8"E	June–August 2014	30 (30)	2.283	0.302	0.362	0.164
	Blanes 41°40′44.5"N 2°48′28.8"E	June–August 2014	37 (37)	2.284	0.296	0.360	0.177
	Xabia 38°47′46.1"N 0°11′01.4"E	May–July 2014	28 (26)	2.255	0.281	0.354	0.207
	Palos (Cabo de Palos) 37°38′05.4"N 0°41′31.6"W	May–August 2014	26 (24)	2.229	0.285	0.350	0.185
	Aguamarga 36°56′20.8"N 1°55′53.1"W	May–August 2014	28 (27)	2.242	0.296	0.352	0.158
	Herradura (La Herradura) 36°43′40.0"N 3°44′07.9"W	July–August 2016	13 (6)	2.224	0.284	0.352	0.193
*Symphodus tinca*	Colera 42°24′26.9"N 3°09′23.8"E	May–June 2015	30 (24)	2.600	0.298	0.356	0.164
	Blanes 41°40′44.5"N 2°48′28.8"E	May 2014	31 (27)	2.588	0.293	0.355	0.175
	Ametlla (L’Ametlla de Mar) 40°52′27.1"N 0°47′42.3"E	May–June 2015	26 (23)	2.597	0.301	0.356	0.155
	Xabia 38°47′46.1"N 0°11′01.4"E	May–June 2015	28 (28)	2.601	0.293	0.356	0.178
	Santa Pola 38°11′27.9"N 0°33′57.2"W	2015	26 (0)	2.592	0.293	0.355	0.173

**Table 2:** Mean values and standard deviation of individually based environmental and phenotypic variable values by location. No environmental and phenotypic variable is available for Santa Pola in *Symphodus tinca*, since juveniles with otolith information were not sampled in this locality.

Species	Location	Planktonic Larval duration (PLD) (days)	Settlement size (µm)	PLD Growth rate (µm/day)	Hatching date (day of the year)	Surface temperature (ºC)	Productivity (ChlA mg/m^3^)	Turbulence (m)
*Symphodus ocellatus*	Colera	10.1 ± 1.0	45.4 ± 4.6	4.5 ± 0.6	192.8 ± 10.8	21.8 ± 0.4	0.211 ± 0.005	1.40 ± 0.24
	Blanes	9.3 ± 1.0	48.7 ± 6.2	5.3 ± 0.6	193.4 ± 13.5	22.5 ± 0.9	0.164 ± 0.009	0.67 ± 0.18
	Xabia	9.3 ± 1.2	43.3 ± 7.1	4.7 ± 0.6	186.0 ± 15.8	24.1 ± 1.2	0.171 ± 0.004	0.87 ± 0.08
	Palos	8.6 ± 0.8	42.0 ± 3.8	4.9 ± 0.6	184.2 ± 10.2	23.8 ± 0.9	0.163 ± 0.016	0.97 ± 0.08
	Aguamarga	9.3 ± 1.3	43.8 ± 4.8	4.8 ± 0.6	188.6 ± 19.0	23.1 ± 1.9	0.158 ± 0.003	0.83 ± 0.11
	Herradura	9.3 ± 0.8	46.0 ± 5.6	5.0 ± 0.6	229.7 ± 11.7	23.9 ± 0.3	0.261 ± 0.030	0.90 ± 0.22
*Symphodus tinca*	Colera	9.3 ± 0.9	33.0 ± 2.4	3.6 ± 0.4	159.9 ± 4.9	20.1 ± 0.9	0.306 ± 0.039	1.02 ± 0.23
	Blanes	12.4 ± 1.4	40.9 ± 4.0	3.3 ± 0.2	135.7 ± 5.7	16.5 ± 0.1	0.335 ± 0.011	0.72 ± 0.11
	Ametlla	9.0 ± 1.0	35.8 ± 4.0	4.0 ± 0.4	157.0 ± 5.2	22.0 ± 1.3	0.333 ± 0.066	0.52 ± 0.14
	Xabia	9.9 ± 1.1	42.6 ± 4.6	4.3 ± 0.4	149.0 ± 3.6	21.0 ± 0.6	0.201 ± 0.019	0.59 ± 0.10

### Population genomics

We analysed 303 individuals from the two sympatric *Symphodus* species with GBS (Table 1, Fig. 1). We obtained a mean (± SD) of 2.6 ± 0.8 and 2.6 ± 0.9 million reads per individual for *S. ocellatus* and *S. tinca,* respectively. After filtering, we found 3978 polymorphic haplotype loci and 5123 SNP loci for *S. ocellatus* and 5276 polymorphic haplotype loci and 6833 SNP loci for *S. tinca*. The mean depth per locus was 19.5 ± 11.4 for *S. ocellatus* and 19.5 ± 11.3 for *S. tinca*. The maximum number of alleles per haplotype locus was 17 for *S. ocellatus* and 15 for *S. tinca*. Observed and expected heterozygosities per locality were similar in the two species (Table 1).

**Figure 2:**
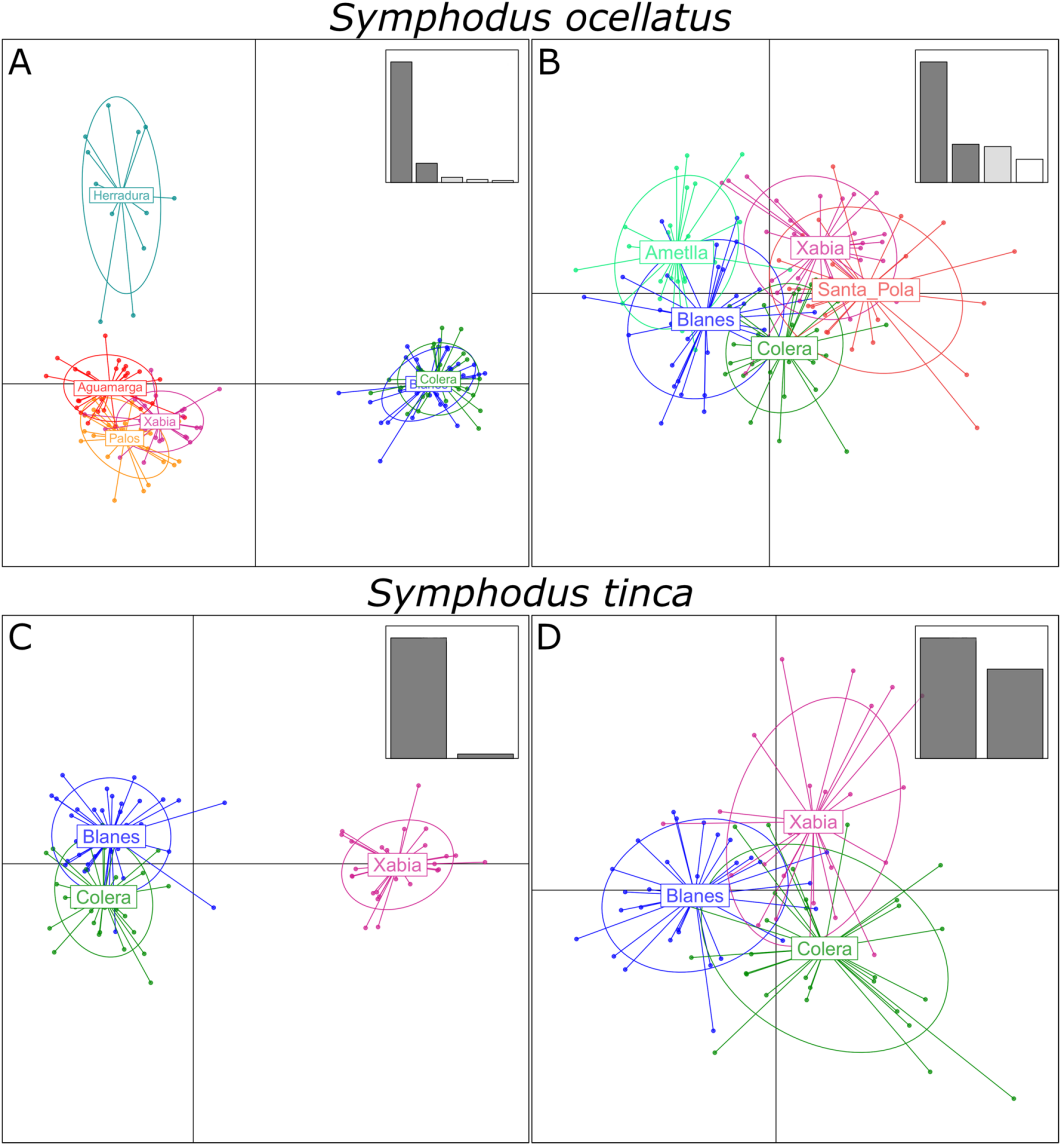
Discriminant Analysis of Principal Components (DAPC). DAPC plots with DA eigenvalues (top right corner) using all analysed localities for each species (**A**,**B**) or only the localities sampled in both species (**C**,**D**).

For *S. ocellatus*, pairwise F_ST_ values (Table S3) as well as the DAPC plot (Fig. 2) and k-means clustering suggest the same structure identifying three groups. One group was formed by the northern localities (Colera – Blanes), another included the localities between the IC and AOF oceanographic discontinuities (Xabia, Palos and Aguamarga) as a central group, and the last one was constituted by the southernmost locality (Herradura) sampled in the Alboran Sea (Fig. 1). It is worthwhile noticing that Herradura clusters with the localities of the central group in the first axis. However, we did not find isolation by distance with a Mantel test (r = 0.45, *p *value = 0.08). For *S. tinca* no clear structuring was detected (Fig. 2, Table S4), and only two significant F_ST_ values were found, when Santa Pola was compared to Blanes and to Ametlla. When comparing only the three localities sampled in both species, we observed similar groupings in the DAPC analysis as when considering all sampled localities, with a clear effect of the IC discontinuity genetically differentiating Xabia from the northern localities in *S. ocellatus* but not in *S. tinca* (Fig. 2). As for *S. ocellatus* we did not find isolation by distance with a Mantel test (r = − 0.38, *p *value = 1).

In the outlier analysis with Bayescan after correcting for multiple comparisons, we identified 95 outlier haplotype loci potentially under positive selection for *S. ocellatus* and 5 for *S. tinca* (Tables S5 and S6).

### Genomic associations to phenotypic and environmental variables

**Figure 3:**
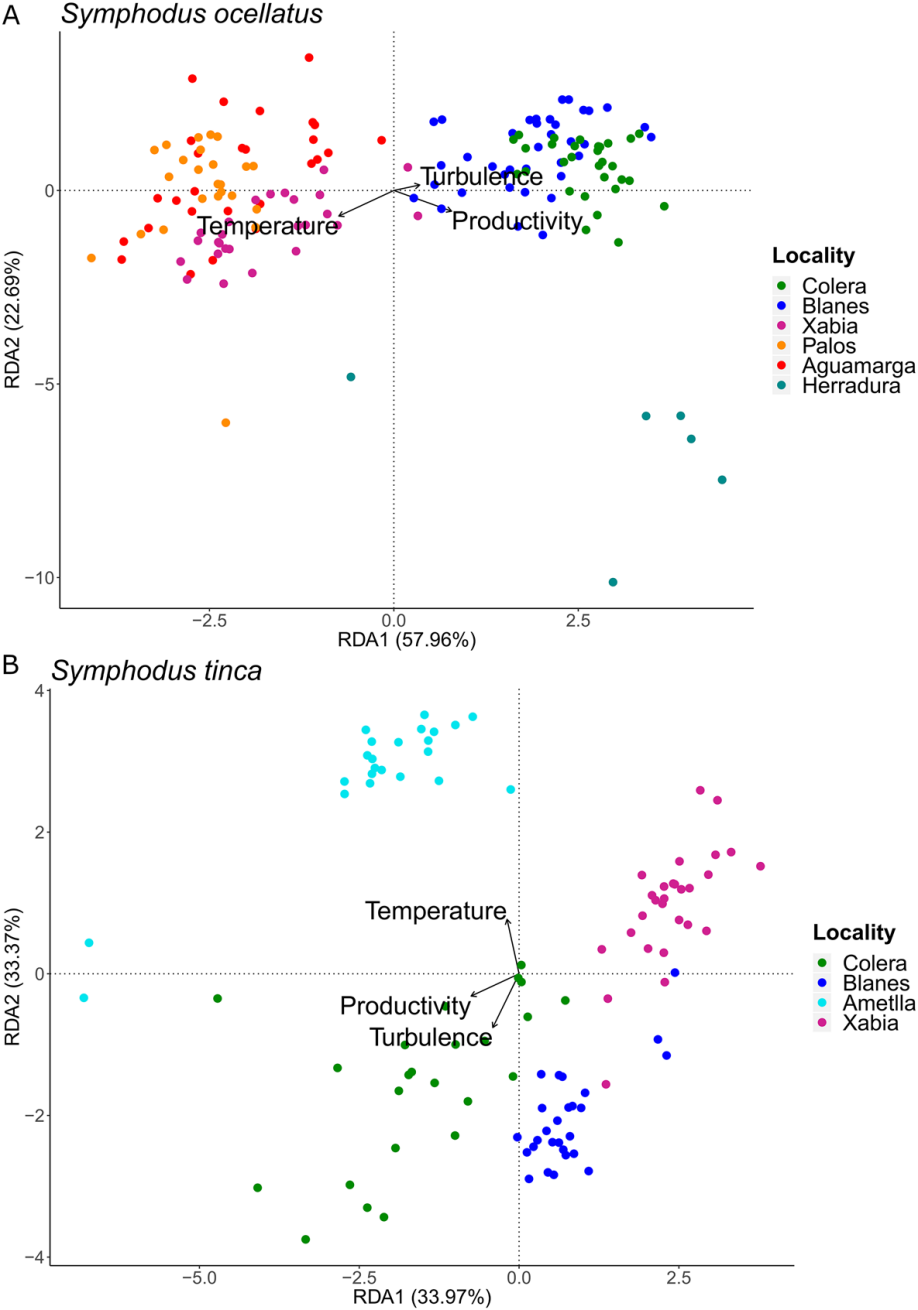
Redundancy analysis (RDA). RDA performed with individual environmental data and SNPs at all loci. Coloured points represent individual fish coded by locality. Vectors indicate environmental predictors of the two first RDA components. (**A**) *Symphodus ocellatus* and (**B**) *S. tinca*.

The variance partition analysis using the SNP loci datafile showed a small contribution of environmental and phenotypic variables in explaining the whole genomic variance in both species, although higher for environmental variables (Table S7). The redundancy analysis (RDA) for *S. ocellatus*, using the individual environmental values and the genotypes from the SNP loci datafile, identified two clusters of individuals along the first axis, explaining 58.0% of the variation (Fig. 3). All individuals of the central group (Xabia, Palos and Aguamarga) clustered together and were positively associated with temperature and negatively associated with productivity while those from the other three localities (Colera, Blanes and Herradura) clustered also together but presented the opposite trend. Overall, 155 haplotype loci had SNPs significantly correlated with environmental predictors (Table S8) with only one locus (20878) being associated to more than one predictor, although with different SNPs (Table S5).

For *S. tinca*, the two axes of the RDA explained 67.4% of the variation and separated the individuals by locality (Fig. 3). The first axis was associated mainly to productivity while the second axis was associated to temperature and turbulence with a different sign. Consequently, Ametlla and Xabia were associated to higher temperature and low turbulence, while Xabia and Blanes seemed to have less productivity. Overall, 166 haplotype loci had SNPs significantly correlated with environmental predictors (Table S8) with only three haplotype loci (12921, 24436, 9609) associated to more than one predictor, although in all cases with different SNPs (Table S6) as in *S. ocellatus*.

The GWAS using as covariates the two first components of the MDS analyses (GWAS-Cov) identified for *S. ocellatus* only one SNP associated to the first environmental component (Table S8). However, when performing the analysis without covariates 207 haplotype loci presented SNPs significantly associated to environmental and phenotypic variables. Most of the SNPs were associated to environmental variables, particularly productivity and temperature and 63 of them were related to more than one variable (Table S5). For phenotype-genotype associations, 21 SNPs were associated to the composite phenotypic variable PC2, positively related to PLD and settlement size (Table S2). In the case of *S. tinca*, GWAS-Cov found six SNPs with associations to phenotypic and environmental variables (Table S8). The same SNPs were associated to the same variables when covariates were not used in the GWAS, with the exception of SNP 12307_17 where the association to EC3 was not detected when using covariates (Table S6).

**Figure 4:**
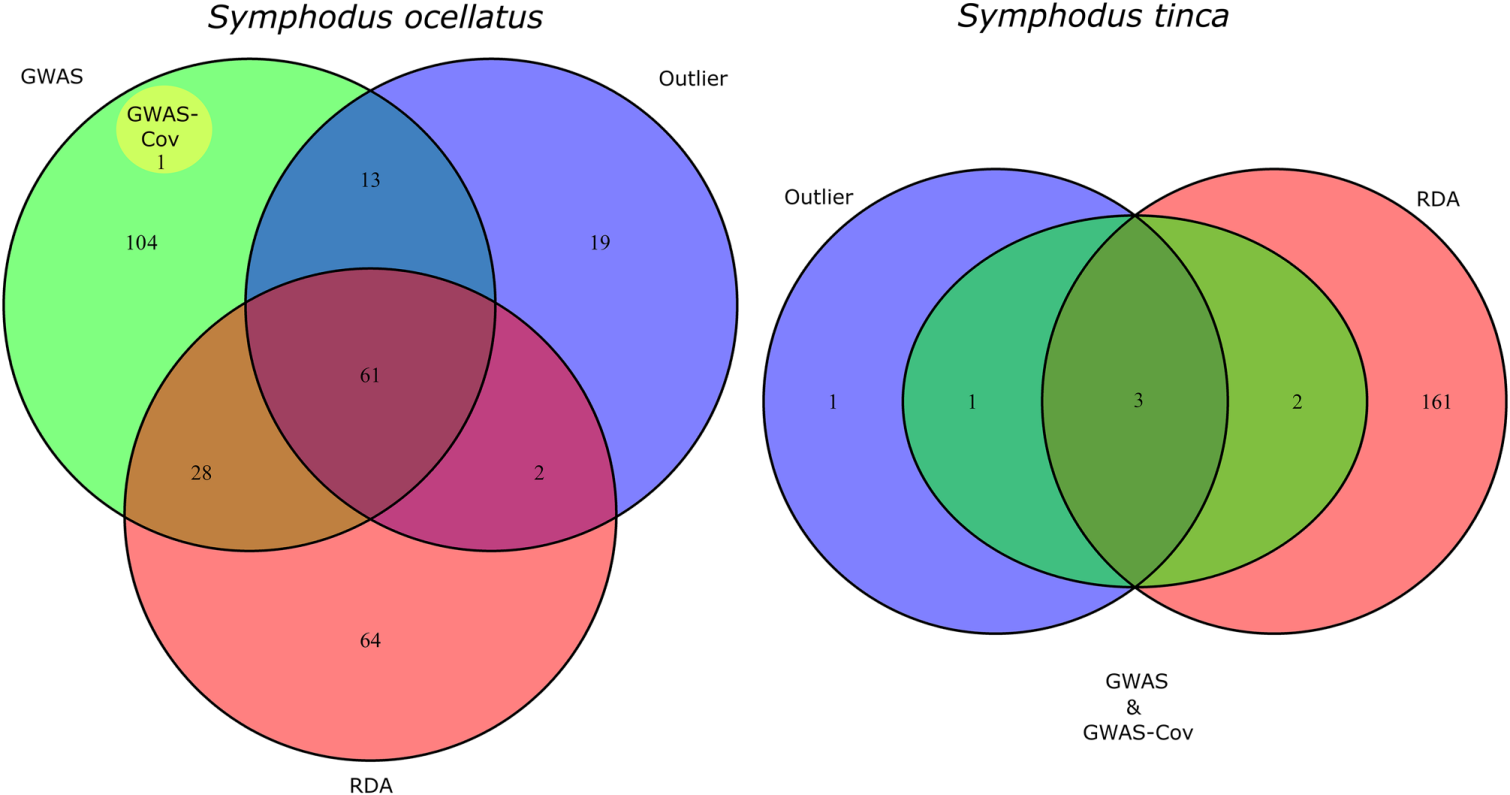
Venn diagram of the candidate haplotype loci showing significant values with different methodologies. Haplotype loci were considered to have a significant value for RDA and GWAS if containing SNPs with significant values for these analyses. Blue: outlier loci; Green: loci associated to environmental and/or phenotypic variables according to GWAS; Yellow: GWAS loci with the two first MDS components as covariates (GWAS-Cov); Pink: loci associated to environmental variables by RDA.

**Figure 5:**
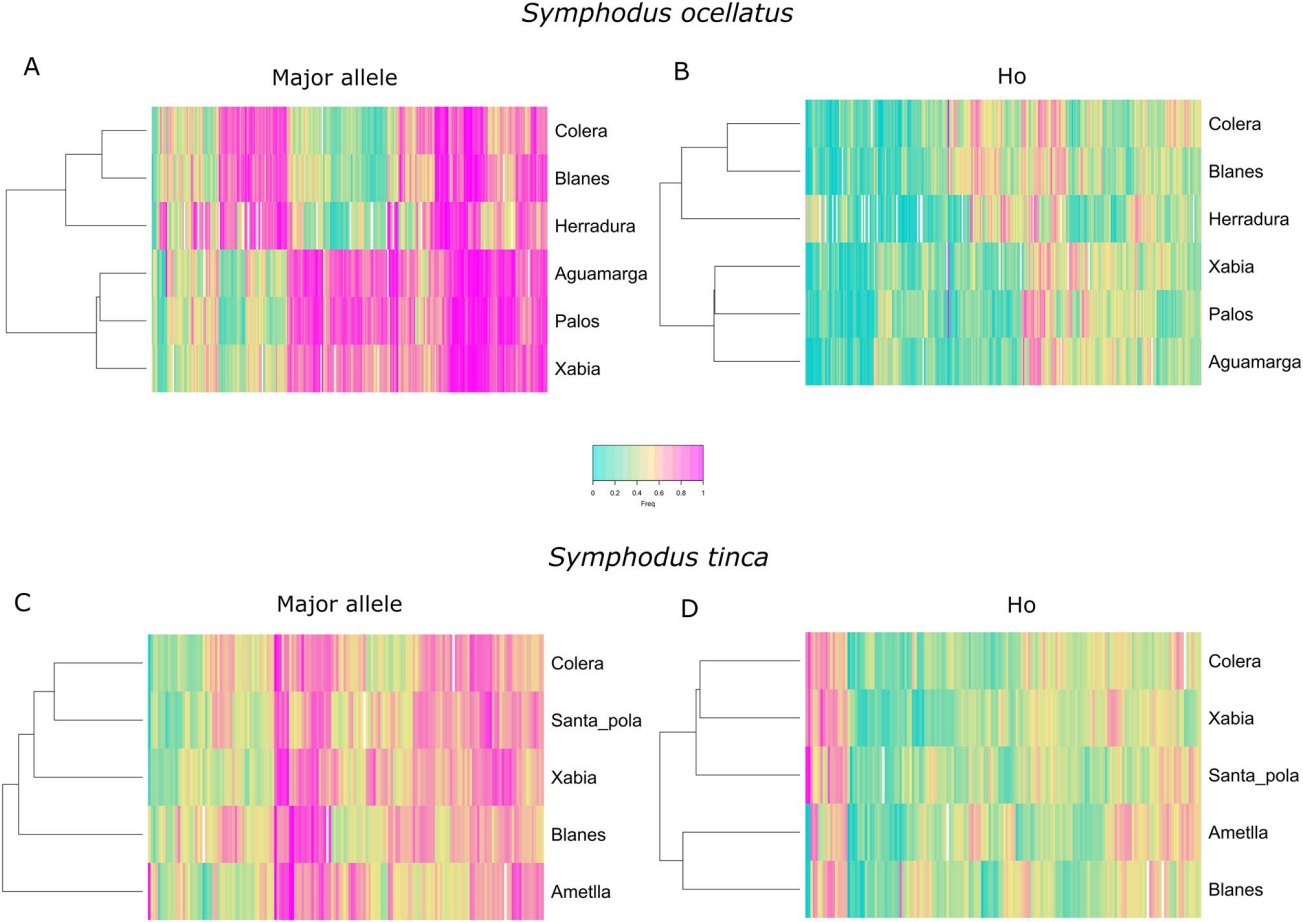
Haplotype loci values showing significant associations. Dendrogram and heatmap grouping the localities based on all candidate loci showing significant values with the different methodologies and using the values from the haplotype loci dataset: *Symphodus ocellatus* (**A**) frequency of the major allele and (**B**) observed heterozygosity. *Symphodus tinca* (**C**) frequency of the major allele and (**D**) observed heterozygosity.

Combining all methodologies (RDA, GWAS-Cov, GWAS and Outliers), a total of 292 and 168 haplotype loci were identified as potentially candidates to selection in *S. ocellatus* and *S. tinca*, respectively (Fig. 4). A low number of loci were shared by all methodologies being larger in *S. ocellatus* which presented higher population differentiation. The percentage of loci with SNPs associated to environmental predictors differed between the two species. Similar numbers of loci were associated to each environmental variable for *S. tinca*, although slightly larger to turbulence (Figure S2). However, for *S. ocellatus* most loci were associated to temperature and productivity. We used all candidate loci to cluster localities based on their observed heterozygosities and the frequency of their major allele from the haplotype loci dataset. In *S. ocellatus* both heatmaps clustered Colera and Blanes, the two northernmost localities, with Herradura, the southernmost locality (Fig. 5). Similar groupings were also observed when only loci related to temperature and productivity were used but differed for loci associated to turbulence (Figure S3). In *S. tinca* different population clustering was observed depending on the variable used and the SNPs associated to different environmental predictors (Fig. 5, Figure S4). Nonetheless, in both species, contrasting frequencies of heterozygotes and major alleles were found among localities (Fig. 5).

**Figure 6:**
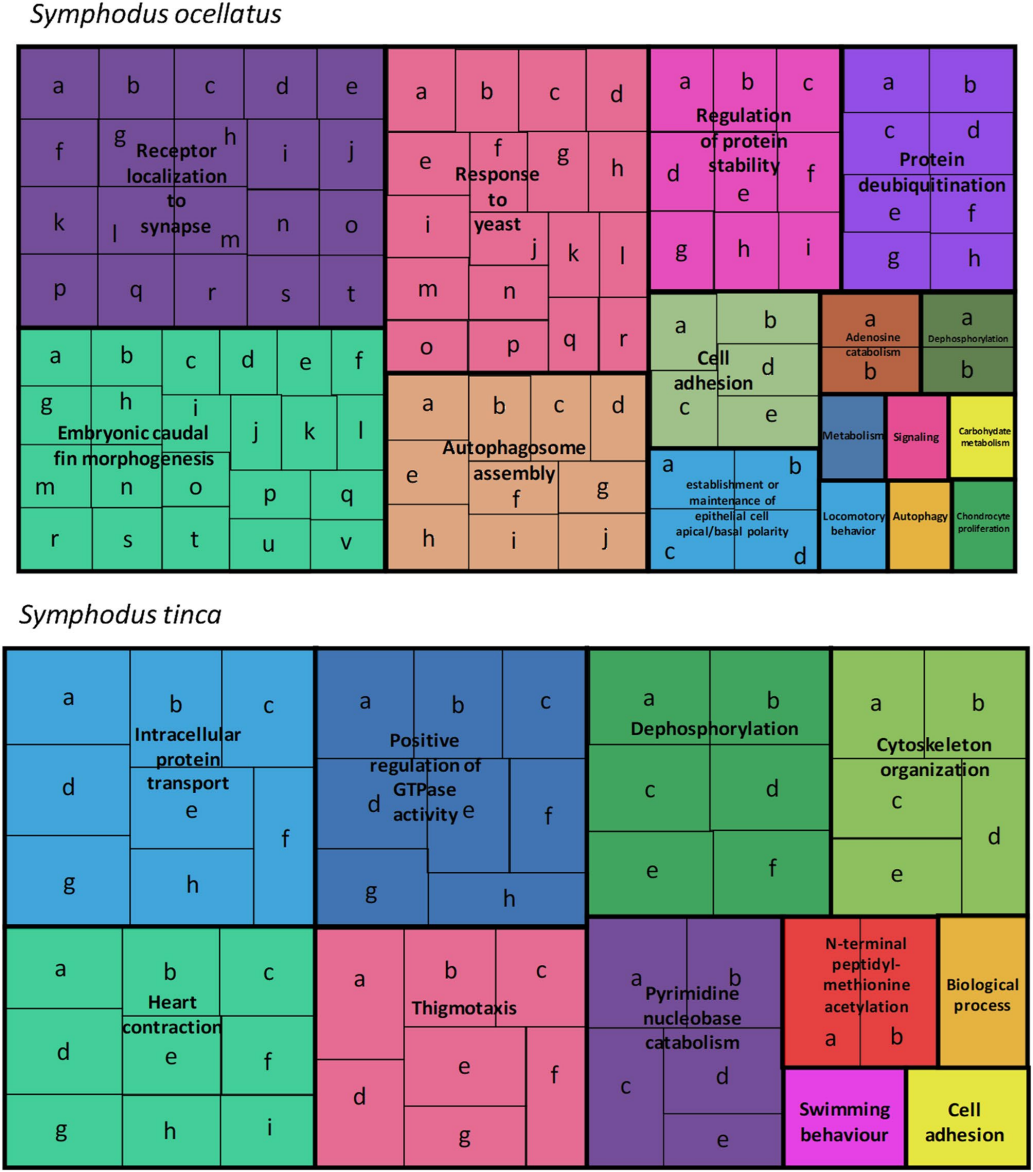
Gene ontology treemap of the candidate genes to selection combining all methodologies in *S. ocellatus* and *S. tinca*. The box size correlates to the –log10 p-value of the GO-term. Boxes with the same colour can be grouped together as correspond to the same upper-hierarchy GO-term which is found in the middle of each box. Description of letters and detailed information of the treemap can be found in supplementary tables S9 and S10.

The Blast of all loci potentially under selection against the Ensemble database provided 172 hits for *S. ocellatus*, 93 of them inside genes (Table S5) and for *S. tinca* 103 loci had a blast hit, 43 of them inside genes (Table S6). The compilation of the biological functions using the GO terms in REVIGO showed that the genes identified in *S. ocellatus* and *S. tinca* had different functions (Fig. 6). On one hand, *S. ocellatus* genes were associated to embryonic caudal fin morphogenesis, receptor localisation to synapse, response to stimuli, protein stability, autophagosome assembly and cell adhesion (Fig. 6). On the other hand, *S. tinca* genes were associated to intracellular protein transport, heart contraction, thigmotaxis and swimming behaviour, regulation of GTPase activity, dephosphorylation, nucleobase catabolism and cytoskeleton organisation (Fig. 6). Furthermore, within each species the genes associated to each of the environmental predictors showed different biological functions (Figure S5 and Figure S6).

## Discussion

The interaction of different evolutionary forces, such as dispersal and selection, defines the actual distribution of the genomic diversity within species^4,22^. We suggest that differences in environmental and phenotypic variables at early-life stages can produce contrasting genomic response that can be revealed by comparative studies on closely related species. Our two studied species presented very contrasting patterns of genetic structure, nonetheless the use of several methodologies for assessing signals of selection revealed multiple loci associated to environmental and phenotypic variables in both of them. Our individual level approach integrating genomic data with environmental and phenotypic information was fundamental for identifying the factors and processes affecting population structuring.

### Population structure and timing of reproduction

Our genome-wide analysis of the two *Symphodus* species, *S. tinca* and *S. ocellatus*, showed different levels of connectivity across the same environmental gradient. Two contrasting patterns are found despite their potentially similar dispersal capabilities, as both species build nests with algae^23,24^ and present similar early-life traits such as planktonic larval duration (PLD), size at settlement and growth rate^25^. However, they have different life span and reproductive season^26,27^. If some adults move to neighbouring areas, species with longer life span such as *S. tinca* could present more connected populations than its congener *S. ocellatus* that has a shorter life span^28^. Adults are considered to be sedentary and remain close to their settling area^26^ and thus other variables such as the time of reproduction could influence population structure.

In *Symphodus ocellatus*, we found a strong population structure and a clear effect of the two main oceanographic barriers in the area, the Ibiza channel and Almeria-Oran front, genetically clustering our sampling locations in three groups. These two oceanographic barriers seem to reduce gene flow across a great variety of taxa with different dispersal capabilities^29^. On the contrary, we observed very low genetic differentiation for *S. tinca* and a null effect of the Ibiza channel, in agreement with a previous study using microsatellite loci^30^. Seasonal variation in the reproductive and larval periods could probably influence the effect of oceanographic fronts on connectivity. For instance, the Ibiza channel although presenting inter-annual variations is known to break more often in spring than in summer^31,32^ thus promoting connectivity for species reproducing in this season^16,33^. In the western Mediterranean, in spring there are more extreme turbulence values and higher frequency of unpredictable energy fluxes and episodic events than in summer^34–36^ which could enhance connectivity. Contrarily to the results we found in the western Mediterranean, a significant population genomic structure was unveiled for *S. tinca* in the Adriatic Sea^10^. Interestingly, during the reproductive season of *S. tinca*, lower turbulence is observed in the Adriatic Sea in comparison to the western Mediterranean^34^.

Some fronts, such as the AOF, seem to reduce connectivity for many marine species across all taxa, although many other species seem not to be influenced^29,37^. This could be attributed to temporal differences. Annual variation in the current pattern across the oceanographic fronts seems to account for genetic differentiation in neighbouring locations in crabs and sea urchins^33,38,39^. Seasonal variation in the hydrodynamic conditions across the fronts^31,40^ could also differentially affect population structure in different species according to their reproductive season, as found in the present study. Therefore, we hypothesize that more stable oceanographic conditions could reduce genetic connectivity, facilitate long term local adaptation and leave more detectable genomic footprints of selection. Further population genomic studies across the same barriers in a wider number of species considering the reproductive season could allow testing this hypothesis.

### Genomic associations to phenotypic and environmental variables

Local adaptation might be shaping population structure along with connectivity reduction mediated by oceanographic barriers^41^. Different genotypes can be under diverse selective pressures among localities, rendering individual-based information highly valuable. The use of several methodologies was fundamental to capture the signatures of adaptation in species with contrasting degree of population structuring. We found, in both species, small contribution of our studied variables to genomic structuring with the variation partitioning of the SNPs datafile at the individual level. These results confirm that the effect of external factors in genomic structuring is small across the whole genome, but does not preclude to be important in some specific loci. For instance, in *S. ocellatus* the higher population differentiation resulted in a higher number of outlier loci and potential for detecting local adaptation in comparison to *S. tinca*. Nonetheless, for both species of *Symphodus,* the combination of the results of GWAS and RDA revealed a larger number of candidate loci, putatively under selection, as well as the selection drivers of most of the outlier loci.

Redundancy analyses have been successfully used to detect signals of selection by comparing population allele frequencies to their environmental variables^4,6^. As we had individually based environmental data, we assessed how each single individual was distributed along the RDA axes based on its genotype, and according to its environmental predictors^42^. In *S. ocellatus* the individuals were separated in two groups along the first RDA axis, which was explained by surface temperature and productivity*.* This analysis grouped geographically separated localities, that present low temperature and high productivity, thus demonstrating that population differentiation is not only influenced by oceanographic barriers but also respond to environmental selection pressures. For, *S. tinca* individuals were grouped by locality in the RDA analysis, but their clustering was less clear, with temperature, productivity and turbulence having similar impact. Thus, despite the low number of outlier loci and population structure in that species, environmental variables can nonetheless contribute to adaptation. These apparently contradictory results rely on the power of different methodologies to detect signals of selection. The analysis of outlier loci is based on the detection of loci that show divergent patterns of genetic differentiation among localities. Thus, this analysis is highly dependent on the basal levels of population structuring as indicated in previous studies^4,42^. For instance, the abundance of outlier loci in *S. tinca* found in our study area (0.1% of all loci) was lower than that obtained for the same species in the Adriatic Sea (0.5%)^10^, suggesting that a more structured region can favour the power to detect local adaptation by outlier analysis. For this reason, RDA should be also used, as it can detect signals of selection even on conditions of low structuring^42^. Moreover, this approximation can be done both at the individual and population level and can thus be implemented in species where individual based information is difficult to obtain.

The analyses of phenotypic and environmental variables associated to individual genotypes are necessary to unveil candidate regions of the genome determining these traits^3,6^. However, when there is population structure, signals of selection within them can be confounded by either demographic or selective processes among populations^43^. In both *Symphodus* species we identified only a few candidate loci associated to environmental variables by using GWAS after removing the population effect using the two first MDS axis as covariates. This methodology has been suggested to control for hierarchical structure to avoid identifying spurious associations due to population differentiation. However, this approach is very conservative and can underestimate phenotype-genotype associations in loci under local adaptation^4,42^. For instance, in *S. ocellatus*, the high level of population structuring undermines the power to detect selection using GWAS with covariates, while GWAS without covariates and RDA revealed a high number of candidate loci to selection. In the case of *S. tinca*, the low level of population structuring limited the resolution of the two types of GWAS while many loci under selection were found with RDA. This highlights the need to assess selection using different methodologies as the degree of population structuring may underestimate the role of selection depending on the methodology used.

Environmental factors, such as temperature or salinity, are known to impose differential spatial signatures of selection on marine organisms^6,44,45^. The biological functions of the genes associated to the candidate loci of selection in our two *Symphodus* species suggest different mechanisms of adaptation in response to the environmental factors although both of them showed strong influence of temperature and productivity. The importance of these two variables in marine organisms is not unprecedented. Temperature is a crucial factor in many marine organisms as it may impact metabolism, larval survival and development among others^46^. Interestingly, turbulence was found to be more important in *S. tinca* than in *S. ocellatus*. Although the turbulence values can be similar during the reproduction period of both species, the frequency of storms in our study area is much higher in spring than in summer^34,47^. This temporal instability may explain the importance of this variable on the spring breeder *S. tinca* with 40% of candidate loci associated to this factor while only 11% where found to be associated in *S. ocellatus.*

In both species, we found several polymorphisms to be significantly associated with phenotypic and environmental variables, indicating a possible genotype-phenotype-environment interaction. In *Symphodus tinca*, we identified two loci associated to hatching date and also to environmental variables, being one of them related to turbulence and the other to temperature. Hatching date seems to be under selections in several marine species. For instance, in *S. tinca* reproducing females appear to prefer males with nests early in the reproductive season, and hatching success is consistently higher for eggs placed in early nests^48^. In the sunfish, *Lepomis macrochirus*, individuals born early in the season have higher survivorship than those born late^49^. In the peacock blenny, *Salaria pavo*, males select a different reproductive tactic depending of the birth date^50^. For the common triplefin, *Forsterygion lapillum*, more successful males hatch early in the hatching period^51^. Overall, these studies suggest that individuals born early could attain larger sizes at the reproductive season, rank more highly in social hierarchy and have better chances to reproduce. In *S. ocellatus,* 22 loci were related to settlement size or to PC2, highly influenced by PLD and settlement size, and 70% of them were also associated to temperature. A high proportion (50%) of those loci were also associated to productivity and EC1. Size at settlement, and other early-life traits, clearly increase post-settlement survival in some species, e.g. *Pomatomus saltatrix*^52^, *Thalassoma bifasciatum*^53^, *Symphodus* spp.^54^ demonstrating the existence of a size-selective mortality. However, the absence of a consistent trend for selective mortality^53^ and annual variation among settlement intensity, recruitment level and year class strength^55^ suggest an important role of random events on survival. Therefore, natural selection could increase the frequency of alleles favouring reproduction early in the season as well as size at settlement and high stochasticity in survival maintain genetic polymorphism.

In conclusion, the results of this study show that individual-based information combining genomics, environmental and phenotypic values is key to unveil detailed patterns of evolutionary genomic signatures in living organisms and thus to glimpse the potential response to future changes. Despite the study of early life traits through otolith readings is only applicable to bone fishes, the combination of the different methodologies conducted in this paper to assess population structuring and local adaptation is useful for all species with individualized genotypic, environmental and phenotypic data. Furthermore, this approach has demonstrated to be applicable at the population level and in the absence of individual based information^4,6^. Our findings emphasize that candidate regions to local adaptation, mediated by productivity and temperature, can even be detected in species presenting low population structuring. This holistic approach should be considered in scientifically informed management actions since, in spatially and temporally variable environments, as found along the distribution ranges of most species, environmental and phenotypic factors could be imposing previously concealed selective pressures.

## Materials and methods

### Study species

*Symphodus ocellatus* (Linnaeus, 1758) and *Symphodus tinca* (Linnaeus, 1758) live in shallow waters (0–30 m), have similar larval duration (7–13 days) and are considered to disperse only during the larval stages, since their adults exhibit territorial behaviour^26^. These two species are representative components of the rocky, sea-grass beds and soft bottom communities of the Western Mediterranean^56,57^. They have a narrow bathymetric range (< 100 m), are found ubiquitously across the studied area and produce a clear settlement band in their otoliths^25,58^. The reproductive season of *S. tinca* extends during spring, whereas *S. ocellatus* reproduces in summer (Lejeune, 1985; Raventos & Macpherson, 2005b). *S. ocellatus* individuals live near 5 years, reach a maximum length of 12 cm and attain sexual maturity between 1 and 2 years^27^. *S. tinca* individuals live up to 15 years^28^, can reach 44 cm, and attain sexual maturity in 2 or 3 years^27^. Due to the phylogenetic relatedness and shared biological traits and distribution, these species have a high value for a comparative population genomic study.

### Sampling

A total of 162 individuals of *Symphodus ocellatus* (Linnaeus, 1758) and 141 of *Symphodus tinca* (Linnaeus, 1758) were sampled and analysed from six and five different locations respectively along the Spanish Mediterranean coast between 2014 and 2016 (Table 1, Fig. 1). This area contains two oceanographic discontinuities, the Almeria-Oran Front (AOF) and the Ibiza Channel (IC), that can affect connectivity among populations^29,30^. From Santa Pola, adult fin clips were provided by professional fishermen, while for the remaining localities juveniles were captured during recruitment using hand nets. The whole juvenile individuals and adult fin clips were taken and stored in 96% Ethanol. The collection of fish samples met the Spanish and European regulations. According to article 3.1 of the European Union directive (2010/63/UE) from the 22/9/2010, no approval is needed for fish sacrification with the purpose of tissue or organ analyses. Furthermore, the study species *Symphodus ocellatus* and *S. tinca* are not listed in CITES. A map of the sampling localities (Fig. 1) was plotted using the R^60^ packages ‘ncdf4’^61^ and ‘ggplot2’^62^. The colour gradient in the map represents the mean temperature across the larval period of each species in a 2X2 Km grid obtained from Western Mediterranean OPerational forecasting system (WMOP)^63^.

### Phenotypic and environmental variables

At the laboratory, the otoliths (lapilli) of all juveniles were extracted and mounted in an oil droplet on a microscope slide. Age readings and the presence of settlement marks were determined using a light microscope following the standard methodology described in the literature^25,54^. In brief, we counted the age of the individual with the number of otolith marks along the longest radius, and with this information, we inferred the hatch date of each individual (Figure S1). PLD was calculated with the number of otolith marks before the settlement mark. From otolith readings, four early-life traits were considered for each individual: hatching date (expressed as the number of day of the year), pelagic larval duration (PLD, in days), growth rate during the pelagic larval duration (increment in otolith size in µm divided by PLD) and otolith settlement size (in µm). We measured the otolith width six times and used their mean to minimize measurements errors.

For each individual, the hatching date and its PLD value were used to determine the exact time of its pelagic period, over which the individual-based environmental variables were averaged. Three environmental variables were considered: two from remote sensing, surface temperature (in ºC) and productivity (measured as Chlorophyll a concentration (ChlA) in mg·m^-3^), and one from modelling, significant wave height (in meters) as a proxy for turbulence. The water surface temperature was obtained from SOCIB from a daily gridded dataset with a resolution of 0.02 degrees (Balearic Islands coastal ocean observatory and forecasting system, https://www.socib.eu/). Productivity data used in this paper were produced with the Giovanni online data system, developed and maintained by the NASA GES DISC^64^, obtaining monthly mean values of ChlA with a spatial resolution of 4 km. Significant wave height was obtained from SIMAR model from the Spanish state ports agency, with hourly values and a spatial resolution of 0,25 degrees. Individual-based environmental and phenotypic variables were only available for individuals with otolith information (Table 1).

In order to evaluate the possible differences of early-life traits between locations, Permutational Multivariate Analyses of Variance (PERMANOVA) were performed for each phenotypic and environmental variable, as implemented in the R function ‘adonis’, from the ‘vegan’ package v 2.5-2^65^. Two principal components analysis (PCA) using ‘dudi.pca’ function from ‘ade4’ package v.1.7-8^66^ were performed for each species after standardizing each variable, one with all phenotypic and the other with all environmental variables. We kept the minimum number of principal components necessary to explain at least 99% of the variance.

### DNA extraction and genotyping

DNA was individually extracted using the QIAamp DNA Mini Kit (QIAGEN) extraction kit, following manufacturer’s instructions. DNA integrity was checked by gel electrophoresis and quantified by NanoDrop. To build individual genomic libraries by GBS, 1.5–3 μg of DNA for each sample were sent to the Cornell University Biotechnology Resource Centre (BRC). DNA was digested with EcoT22I restriction enzyme and ligated to barcode and common adaptors with appropriated sticky ends as in a previous study^10^. For each plate, one blank sample and 95 samples were pooled and cleaned using QIAGEN PCR cleanup kit (following manufacturer’s instructions). An amplification by PCR was applied to the resulting 96plex libraries, using generic primers matching the adaptors. The PCR was made under the following conditions: 5 min at 72ºC, 30 s at 98ºC, 18 cycles of 10 s at 98ºC, 30 s at 65ºC and 30 s at 72ºC and a final extension of 5 min at 72ºC. The PCR product was cleaned with the QIAquick PCR Purification Kit, diluted and single-end sequenced in an Illumina HiSeq 2500 platform at BRC, by using one lane per plate and the HiSeq v4 reagents kit. Individuals from the same locality were distributed in different lanes.

Raw Illumina sequences from all samples were used for genotyping with STACKS vs 1.47 software^67^. Good reads with a barcode were trimmed to 59 bp, excluding barcodes and primers. To construct a catalogue loci two mismatches were allowed between stacks within and between individuals, and a minimal stack depth of three was required. Individual genotypes were outputted both as haplotype loci and SNP loci VCF files. Additional filters were applied using VCFtools vs 1.12^68^. Individual genotypes with a depth below 5X were not considered. Loci with a missingness value higher than 30% were removed from the loci datafiles. Additionally, haplotype loci with the major allele frequency equally or higher than 0.95 (*i.e.* monomorphic at that level) were identified by the R function ‘isPoly’ from the package ‘adegenet’^69^ and removed from the vcf file by VCFtools. For SNP loci, those with a minimum allele frequency (MAF) of 0.05 were also removed from the SNP dataset by VCFtools. Finally, the loci in HW disequilibrium at more than 60% of the sampling sites^10^ were eliminated of the analysis from both the haplotype and SNP loci datafiles. This filter was used step to eliminate possible paralogous loci.

### Population genomic analyses

We used the haplotype loci datafiles for the population genomic analyses. For each locality, observed (Ho) and expected (He) heterozygosities, allelic richness (Ar), F_ST_ pairwise values and their significance (computed by 999 permutations) were obtained by R software^60^ ‘hierfstat’ package vs 0.04–2^70^. A FDR correction for multiple comparisons was applied in all required analyses to estimate the threshold of differentiation^71^. Isolation by distance was evaluated between Euclidean geographic distances and F_ST_ distances with Mantel tests, as implemented by the ‘mantel’ function in the ‘vegan’ R package. Discriminant Analysis of Principal Components (DAPC) were performed retaining a number of PCAs equal to one third of the number of individuals. To detect the optimal number of genetic clusters, we performed K-means clustering from k = 1 to k = 10, using 10^5^ iterations, retaining a number of PCs equal to sample size and compared the results using the Akaike Information Criterion (AIC). The analyses were performed with the R software package ‘adegenet’ and represented with the ‘ggplot2’ package^62^. To identify outlier loci (OAs), we used BayeScan vs 2.1^72^, with individuals grouped by sampling locality. To minimize false positives, 100,000 simulations were run and a prior odd of 10,000 was specified^73^. Those haplotype loci with a q-value (FDR analogue of the p-value) below 0.05 were considered as statistically significant outliers.

We used the SNP datafile for association analysis with environmental (EAAs) and phenotypic (PAAs) variables. We kept all polymorphic SNPs in each locus after filtering. Missing genotypes were imputed by replacing them with the most common genotype across all individuals. We performed a variance partition analysis for the complete matrix of SNPs, the matrix of the three environmental variables and the matrix of the four phenotypic variables using the ‘varpart’ function in the ‘vegan’ R package. We looked for SNPs strongly correlated with environmental variables by using a Redundancy Analysis (RDA) with the SNP loci datafiles following the method described by Forester and co-authors^42^. We used as response variables a genotypic matrix with the SNPs of all loci and the three environmental variables as predictors. All environmental variables were retained because they were not highly correlated (r < 0.75).

A genome-wide association analysis (GWAS) was performed with the linear model implemented in the software PLINK vs 1.9^74^. For each individual, we used its genotype from the SNP loci datafile and its environmental and phenotypic variables as well as the composite PCA variables previously detailed. Moreover, we performed a Multidimensional Scaling analysis (MDS) with the SNP datafile per species with the same software. The effect of genetic differentiation among localities is often removed in associated studies with the use of covariates. Thus, we carried out two association studies with PLINK: one using as covariates the two first components of the MDS (GWAS-Cov), and the other without covariates (GWAS).

For all candidate loci potentially under selection, identified as population outlier loci and/or including SNPs associated to environmental or phenotypic variables, we computed the frequency of the major allele and their observed heterozygosity for the haplotype dataset in each sampling location. Both values were represented in heatmaps and hierarchical dendrograms, using the heatmap.2 function of the package ‘gplots’ vs 3.0.1^75^.

BLAST searches were conducted with the 59 bp sequences of all candidate loci against the *Labrus bergylta* Ascanius, 1767 genome^76^, available in BLASTn search tool of Ensembl website (www.ensembl.org). Sensitivity was set to “Distant homologies” to maximise matches’ length considering the phylogenetic distance between species. A maximum E-value of 10^–3^ was allowed and only matches of at least half of the locus sequence were considered. When sequences matched within a gene, the function of that gene was searched at the UniProt database (www.uniprot.org). For the list of annotated genes their GO-terms were introduced in REVIGO to produce treemaps of the biological functions^77^.

## Supplementary information

Supplementary Figures.

Supplementary Tables.

Supplementary Dataset 1.

Supplementary Dataset 2.

Supplementary Dataset 3.

Supplementary Dataset 4.

## Data Availability

Raw read data from all individuals is available in the NCBI SRA Bioprojects PRJNA646056 (*S. ocellatus*) and PRJNA646057 (*S. tinca*). A list of genotypes of all individuals, and their sampling location, environmental and phenotypic variable values are available as Supplementary Dataset.
