# Supplementary material for: Individual-based population genomics reveal different drivers of adaptation in sympatric fish
Source: Sci Rep. 2020 Jul 29;10:12683. doi: 10.1038/s41598-020-69160-2 (PMC7391720; doi:10.1038/s41598-020-69160-2)
Supplement: Supplementary file 1 — Supplementary Figures. [file 41598_2020_69160_MOESM1_ESM.docx]

**Individual-based population genomics reveal different drivers of adaptation in sympatric fish**

Héctor Torrado^1, 2^ *, Carlos Carreras ^2^, Núria Raventós ^1^, Enrique Macpherson ^1+^, Marta Pascual ^2+^

^1^Centre d’Estudis Avançats de Blanes (CEAB-CSIC), Car. Acc. Cala St. Francesc 14, 17300 Blanes, Girona, Spain.

^2^Department de Genètica, Microbiologia i Estadística and IRBio, Universitat de Barcelona, Av.Diagonal 643, 08028 Barcelona, Spain.

^+^Both authors contributed equally as senior researchers and should be considered to be at the same position

*Corresponding author: [h.torrado@hotmail.com](mailto:h.torrado@hotmail.com)

Running tittle: Genomic responses in congeneric fish

**Supplementary figures**

**
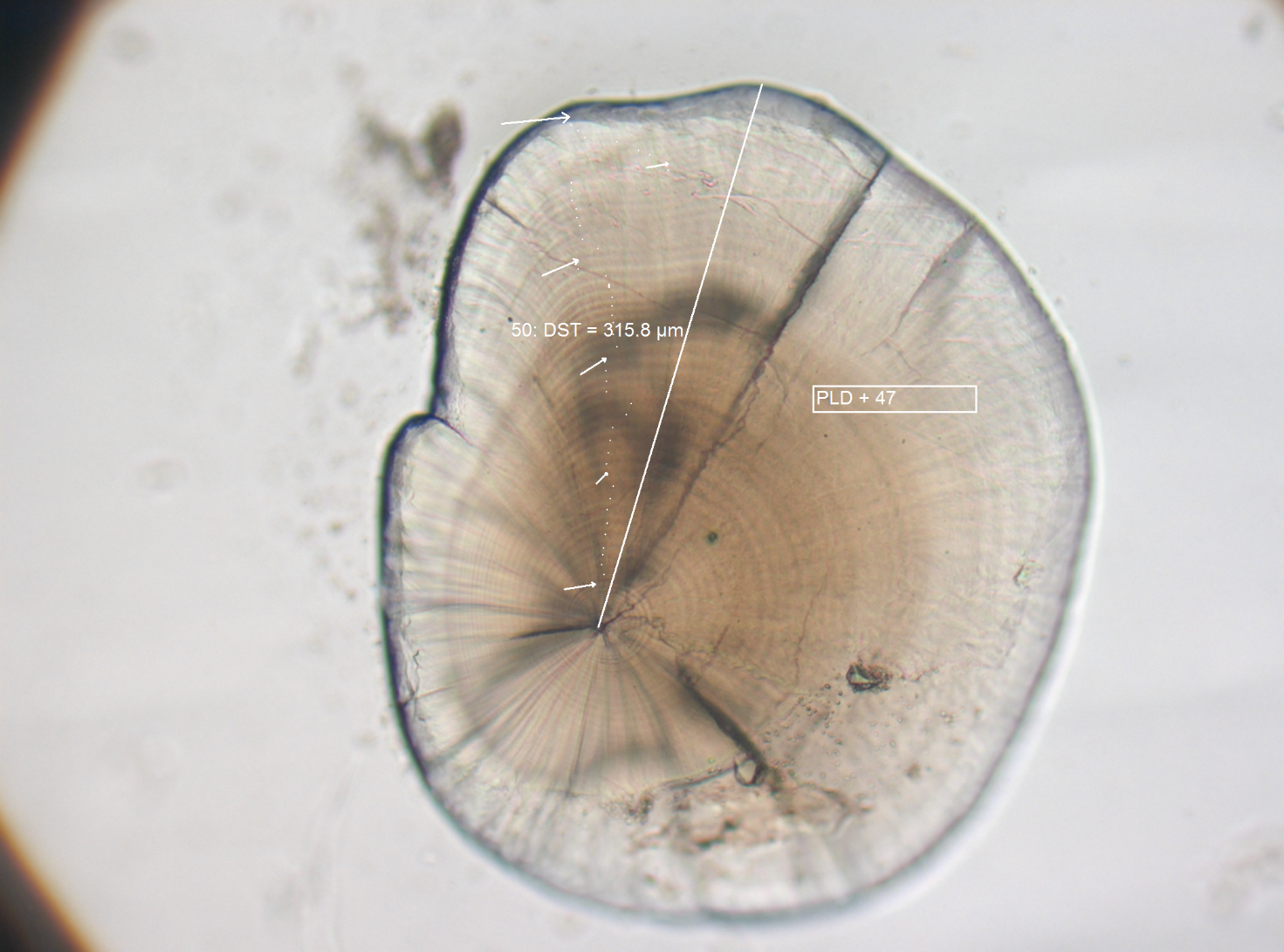
**

**Figure S1: *Symphodus tinca* otolith with dairy increment marks.** The central arrow indicates the settlement ring. White dots and arrows are used to indicate the age of the individual (in the rectangle). The solid line is used to measure the otolith size in µm.

**Figure S2:** **Percentage of haplotype loci with SNPs associated to environmental variables combining the results of different methodologies (RDA and GWAS).** In *S. ocellatus* 54 loci were associated to both temperature and productivity, while only four were associated to more than one of these variables in *S. tinca*.


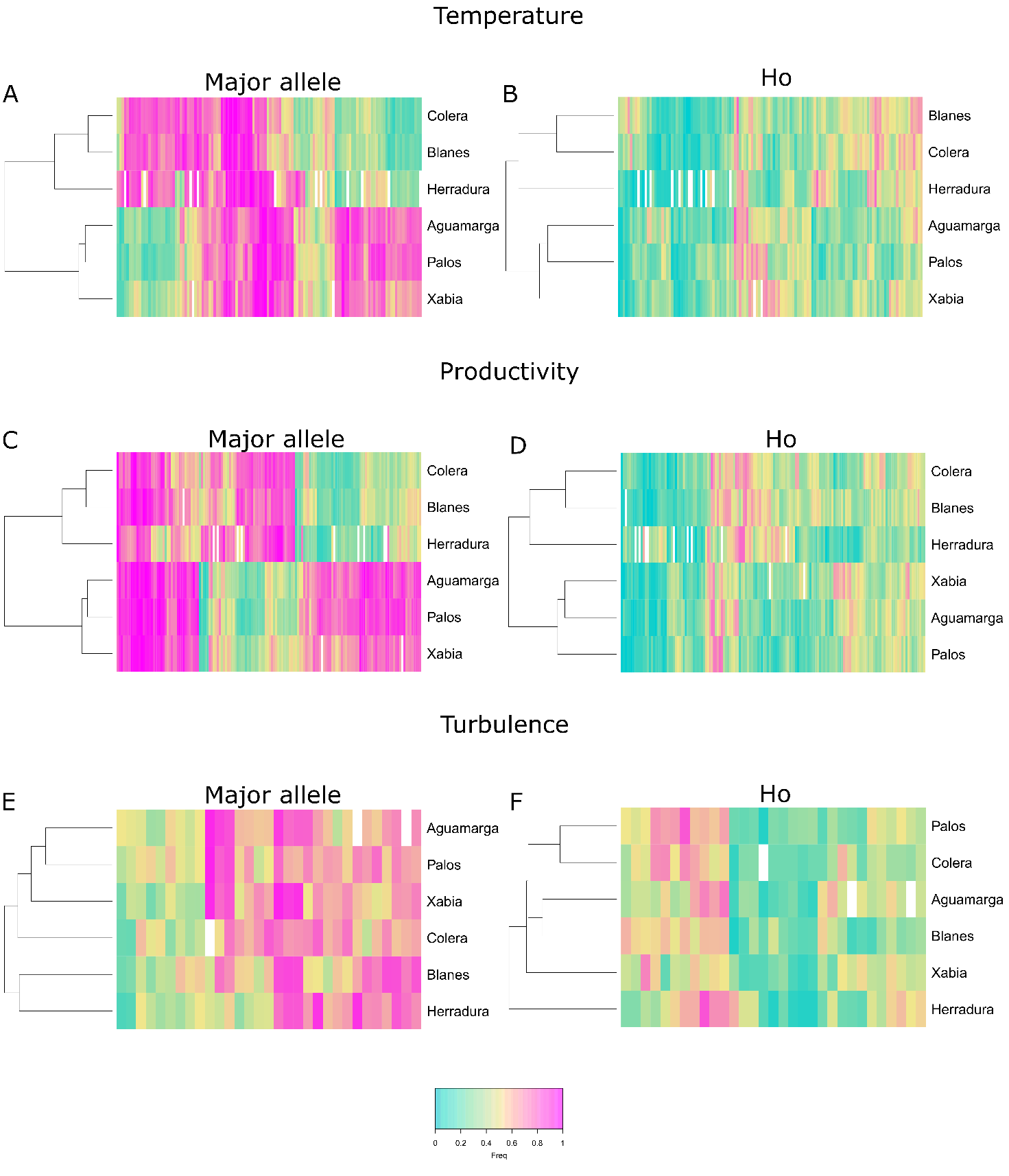
**Figure S3:** **Dendrogram and heatmap grouping the localities based on the frequency of the major allele and observed heterozygosities of loci showing significant values** **in *Symphodus ocellatus*** associated to (A and B) Temperature, (C and D) Productivity and (E and F) Turbulence.


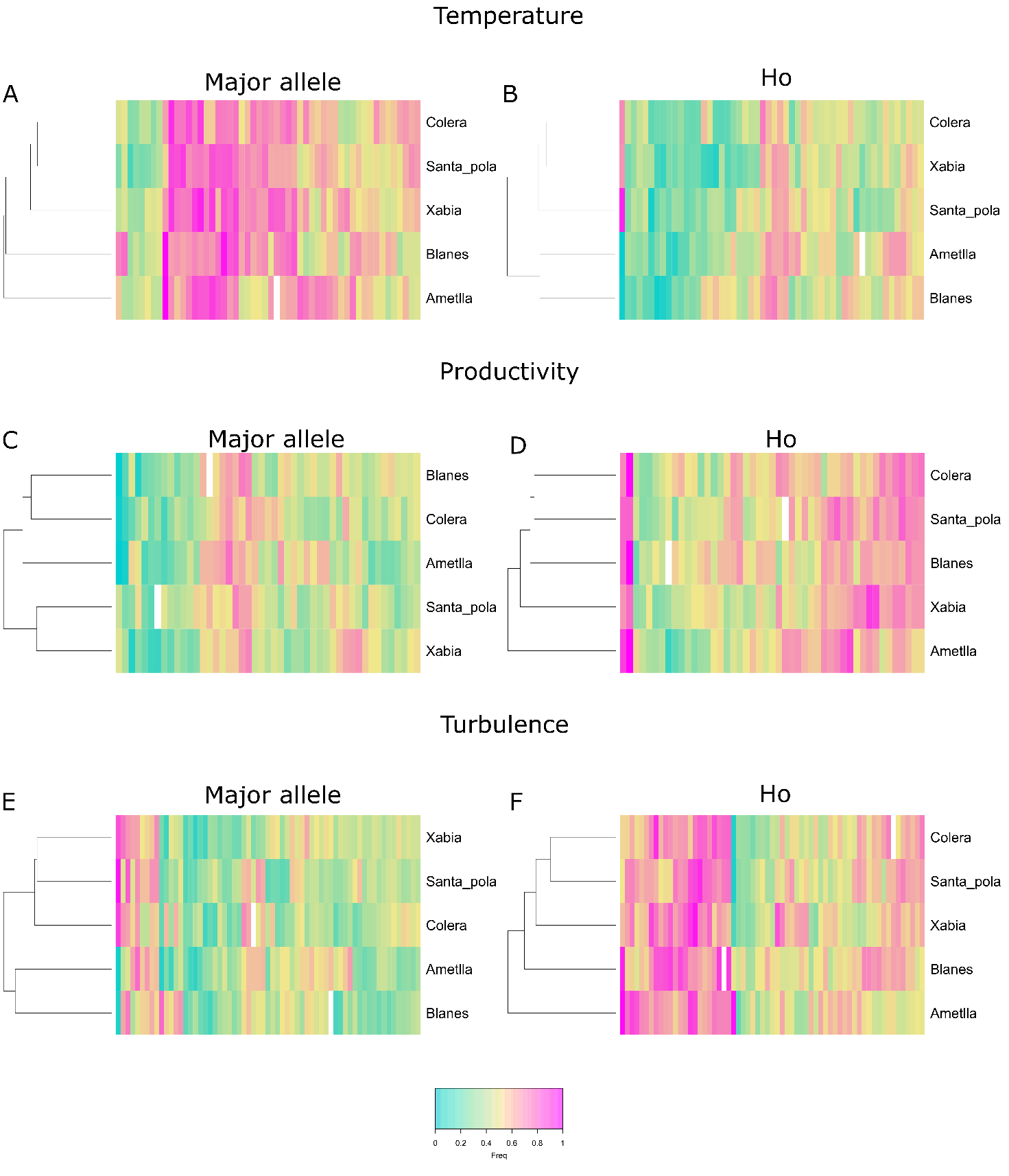


**Figure S4: Dendrogram and heatmap grouping the localities based on the frequency of the major allele and observed heterozygosities of loci showing significant values in *Symphodus tinca*** associated to (A and B) Temperature, (C and D) Productivity and (E and F) Turbulence.


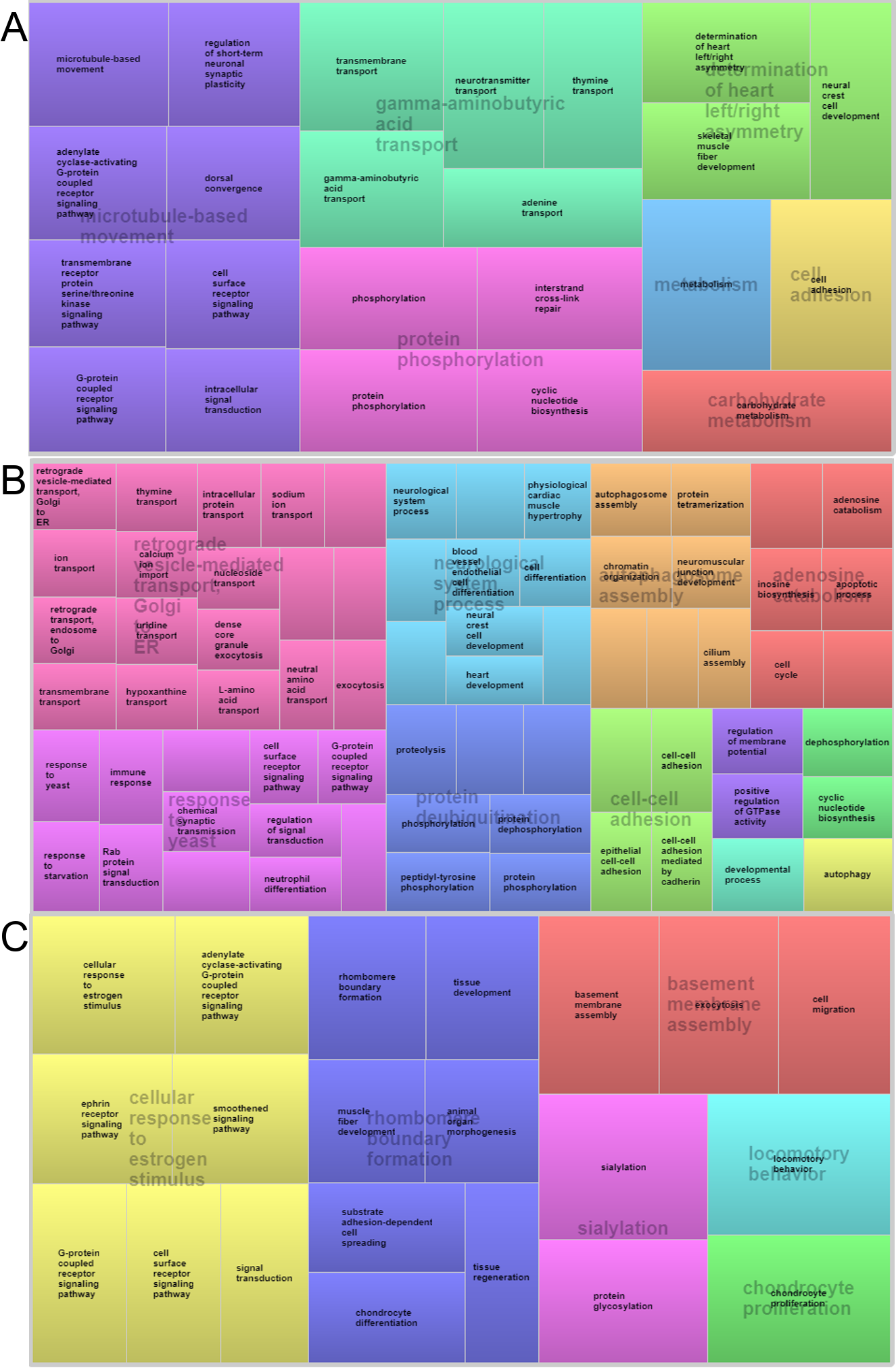


**Figure S5:** **Gene ontology treemap of the candidate genes to selection combining all methodologies in *Symphodus ocellatus*** associated to (A) Temperature, (B) Productivity and (C) Turbulence.


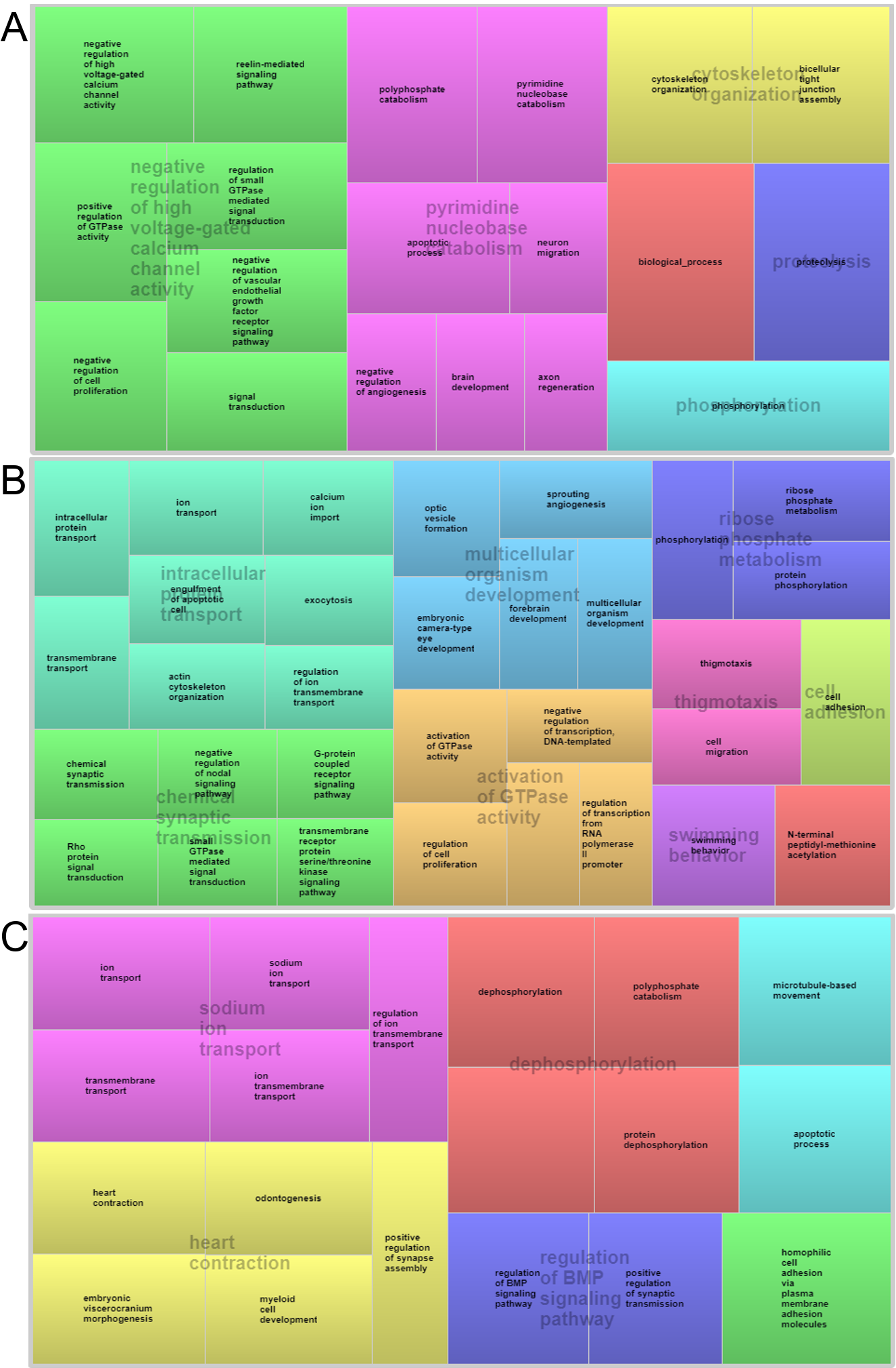


**Figure S6:** **Gene ontology treemap of the candidate genes to selection combining all methodologies in *Symphodus tinca*** associated to (A) Temperature, (B) Productivity and (C) Turbulence.
